# Acute Myeloid Leukemia With Central Nervous System Involvement Following Routine Surgical Procedures: A Bridge Between Surgical, Medical, and Neurological Critical Care

**DOI:** 10.7759/cureus.21245

**Published:** 2022-01-14

**Authors:** Mansur Assaad, Vishal Kumar, Austin Carmack, Apurwa Karki, Daniel Golden

**Affiliations:** 1 Pulmonary and Critical Care Medicine, Guthrie Robert Packer Hospital, Sayre, USA; 2 Trauma and Acute Care Surgery, Guthrie Robert Packer Hospital, Sayre, USA

**Keywords:** medical critical care, surgical critical care, oncologic emergency, general surgery complication, acute myeloid leukemia (aml)

## Abstract

Acute myeloid leukemia (AML) is a rare and aggressive malignancy that can present with a broad range of clinical manifestations. Central nervous system (CNS) involvement is rarely documented and may alter the treatment course and overall prognosis. Although several etiologies have been suggested, the exact mechanism of CNS involvement remains unclear. Furthermore, little is known about the impact of surgical stress on the development of AML. Surgeons should be aware of this potential outcome following surgery, particularly if a leukemoid reaction develops post-operatively, as early detection can prevent delays in appropriate treatment. Further data are needed to better understand the pathogenesis and underlying inflammatory cascades following surgical trauma that possibly contribute to the development of AML.

## Introduction

Acute myeloid leukemia (AML) is an aggressive blood cell cancer that involves clonal proliferation and differentiation of abnormal, malignant hematopoietic precursor cells in the bone marrow [1]. Although AML is the most common acute leukemia in adults, this malignancy represents only roughly 1% of adult cancer-related deaths in the United States [1]. It can arise in patients who have underlying hematological disorders, such as myelodysplastic syndrome, myeloproliferative neoplasms, paroxysmal nocturnal hemoglobinuria, or aplastic anemia [1,2]. AML may also occur due to certain treatments for other malignancies, such as alkylating agents and radiation [1]. Most cases, however, occur as a de novo malignancy in previously healthy patients [1]. Clinical manifestations can vary significantly, and some patients may even be asymptomatic with only laboratory evidence of disease [3]. Common manifestations include anemia, bruising, bleeding, and infections [3]. If the anemia is severe enough, symptoms such as dyspnea, fatigue, and weakness can occur [3]. Lab work will often show significant hyperleukocytosis, anemia, thrombocytopenia, and several metabolic disturbances, including possibly renal dysfunction or tumor lysis syndrome [2,3]. An uncommon and incompletely understood manifestation of AML is central nervous system (CNS) involvement. In fact, a review of three prospective trials by Alakel et al. revealed CNS involvement in less than 1% of 3,261 adults who were newly diagnosed with AML [4]. Several mechanisms for CNS involvement have been proposed, including calvarium bone marrow involvement and extension into bridging veins, involvement of the cerebrospinal fluid (CSF), direct invasion into the brain parenchyma through the blood-brain barrier, and CNS hemorrhage with blood containing blasts [4]. Although patients with CNS involvement may be asymptomatic, a wide array of manifestations may be present depending on risk factors and the overall progression of the disease. For example, symptoms of increased intracranial pressure may result in headaches, lethargy, emesis, seizures, etc. [4]. Additional neurological manifestations can result from leukemic vasculitis, CNS hemorrhage, spinal cord compression, and cranial nerve palsies [4]. Prognosis can be divided into three groups based on risk stratification: favorable, intermediate, and adverse [1,3]. This is mainly determined based on cytogenetic profile and underlying molecular abnormalities [1,3]. Several known risk factors for poor outcomes in adults with AML exist, including age >60, smoking, poor performance status, prior myelodysplastic syndrome or other hematological disorder, prior exposure to cytotoxic agents or radiation, and certain genetic mutations and karyotypic abnormalities [5]. There is very little data in the literature regarding surgical stress as a risk factor for the development of AML. Herein, we present two interesting cases of AML manifesting as altered mental status following routine surgical procedures, both of which presented within one week of each other.

## Case presentation

Case #1

**Figure 1:**
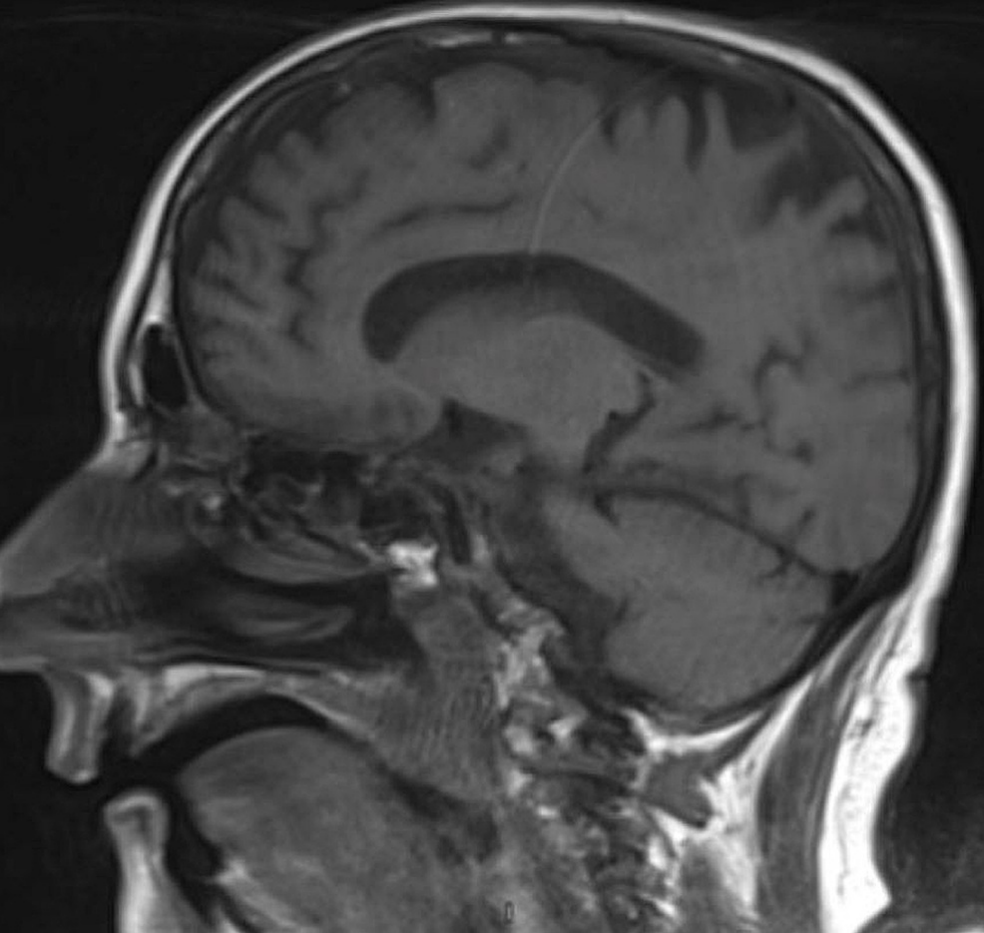
Sagittal T1-weighted magnetic resonance images of the brain showing low-intensity bone marrow signaling, consistent with bone marrow replacement or bone marrow proliferation disorder.

A 66-year-old man with a past medical history of hypertension, hyperlipidemia, obesity, and bladder cancer receiving intravesical Bacillus Calmette-Guerin (BCG) presented to the emergency department with acute abdominal pain and progressive confusion. Per the patient’s spouse, the patient developed progressively worsening weakness and functional status starting five days prior to presentation. The patient did not answer questions appropriately or follow any commands. He was intermittently agitated at times, but still maintaining his airway. Due to the presence of abdominal pain and new leukocytosis on lab work with white blood cell (WBC) count of 20 K/µL, a computed tomography (CT) of the abdomen and pelvis showed findings concerning acute appendicitis with thickening, inflammation, and some localized mesenteric adenopathy. CT of the head was also performed, which showed no acute intracranial abnormality. Additional lab work on presentation showed mild anemia with a hemoglobin concentration of 12.1 g/dL, normal platelet count of 247 K/µL, and new acute kidney injury with a creatinine of 1.8 mg/dL. Blood urea nitrogen (BUN) was normal at 15 mg/dL. The remainder of the lab work, including blood cultures, urine culture, and hepatic function tests were within normal limits. Surgical consultation was obtained, and the patient was taken urgently to the operating room for laparoscopic appendectomy. Intraoperatively, the visualized appendix was mildly inflamed without evidence of perforation. The distal appendiceal tip was thin; however, the proximal base was dilated and hard with surrounding inflammation, concerning for a possible underlying appendiceal or colonic tumor. Dissection of the appendiceal base was difficult due to the presence of significant peri-appendiceal and peri-cecal lymphadenopathy. The procedure was completed without complication. Final pathology showed fibrous obliteration of the entire appendix with no evidence of acute or chronic inflammation. Postoperatively, the patient was managed in the surgical intensive care unit (SICU). He was persistently encephalopathic during his post-operative course despite treatment of suspected sepsis with broad-spectrum antibiotics. At that time, there were no significant electrolyte abnormalities to explain the patient’s altered mental status. On postoperative day (POD) 4, an acute increase in serum sodium was noted to a maximum of 161 mmol/L. Additional lab work showed a decreased urine osmolality of 158 mosm/kg and elevated urine sodium of 60 mmol/L. Given these findings and recent surgical stress, the patient was managed as having central diabetes insipidus. After appropriate treatment with desmopressin, the serum sodium normalized at an appropriate rate and was maintained in adequate range with free water flushes via a nasogastric tube. Given the persistent encephalopathy, central diabetes insipidus, and known history of malignancy, a lumbar puncture was performed to assess for a possible paraneoplastic syndrome affecting the CNS and to rule out septic meningitis. CSF cell count revealed only three nucleated cells. CSF cultures, including routine and mycobacterial, were negative. The CSF cryptococcal antigen, herpes type 1/2 polymerase chain reaction (PCR), and West Nile antibody panel were unremarkable. Magnetic resonance imaging (MRI) of the brain showed a normal pituitary gland and brain parenchyma; however, there was diffuse low-intensity bone marrow signal on T1 weighted images, concerning bone marrow replacement or bone marrow proliferation disorder (Figure 1).

**Figure 2:**
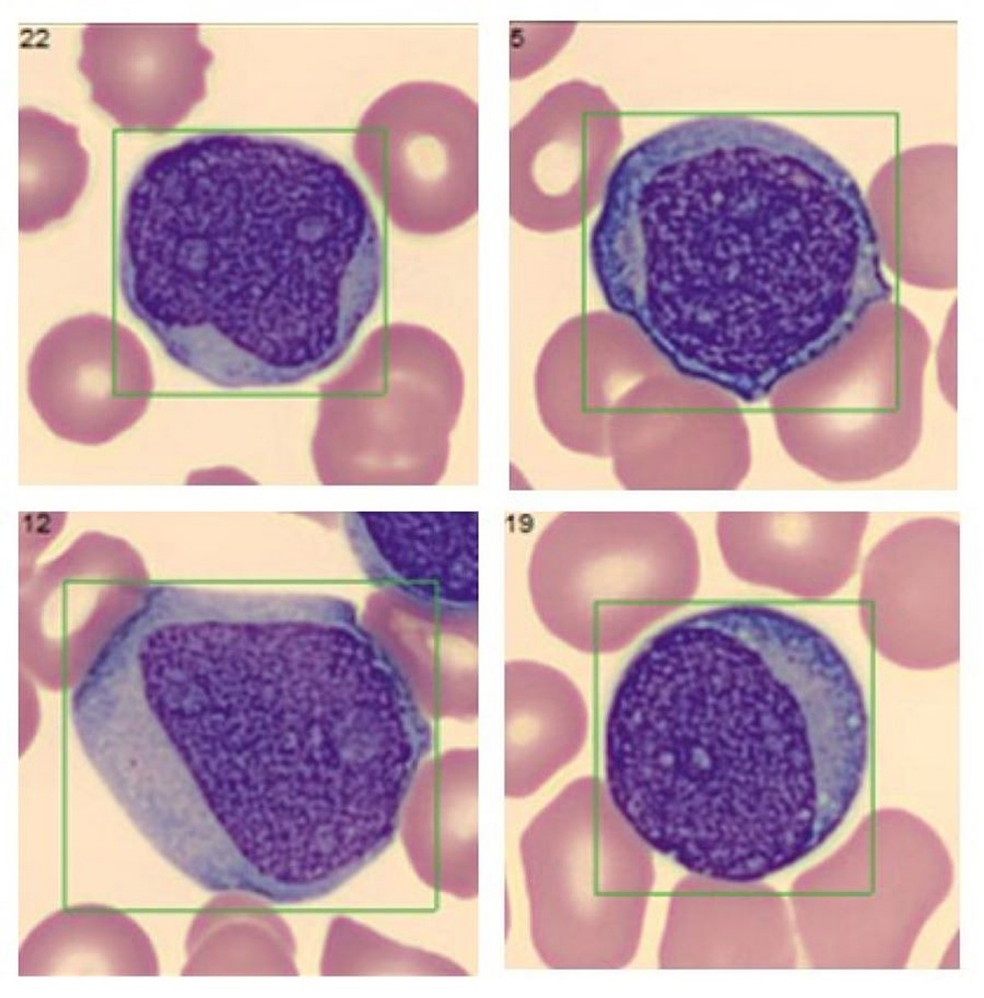
Peripheral smear showing blast cells.

At this point, the patient’s encephalopathy had progressed significantly further. His Glasgow Coma Scale (GCS) was less than 8, and he was no longer able to maintain his airway or follow any commands. He was intubated for airway protection, and ventilator settings were set in accordance with a lung-protective ventilation strategy. On POD 7, lab work was significant for a dramatic increase in WBC count to 55 K/µL, progressive anemia with a hemoglobin concentration of 6.7 g/dL without evidence of bleeding, and a decrease in platelet count to 62 K/µL. Given this new hyperleukocytosis, a peripheral smear was obtained, which revealed 22% blast cells (Figure 2) as well as Auer rods.

Flow cytometry from peripheral blood later confirmed the diagnosis of AML. Urgent hematology/oncology consultation was obtained, and the decision was made to transfer the patient to an outside facility for a higher level of care. Despite this, the patient developed acute respiratory distress syndrome (ARDS), and the exact etiology was never determined. The patient died one week later.

Case #2

**Figure 3:**
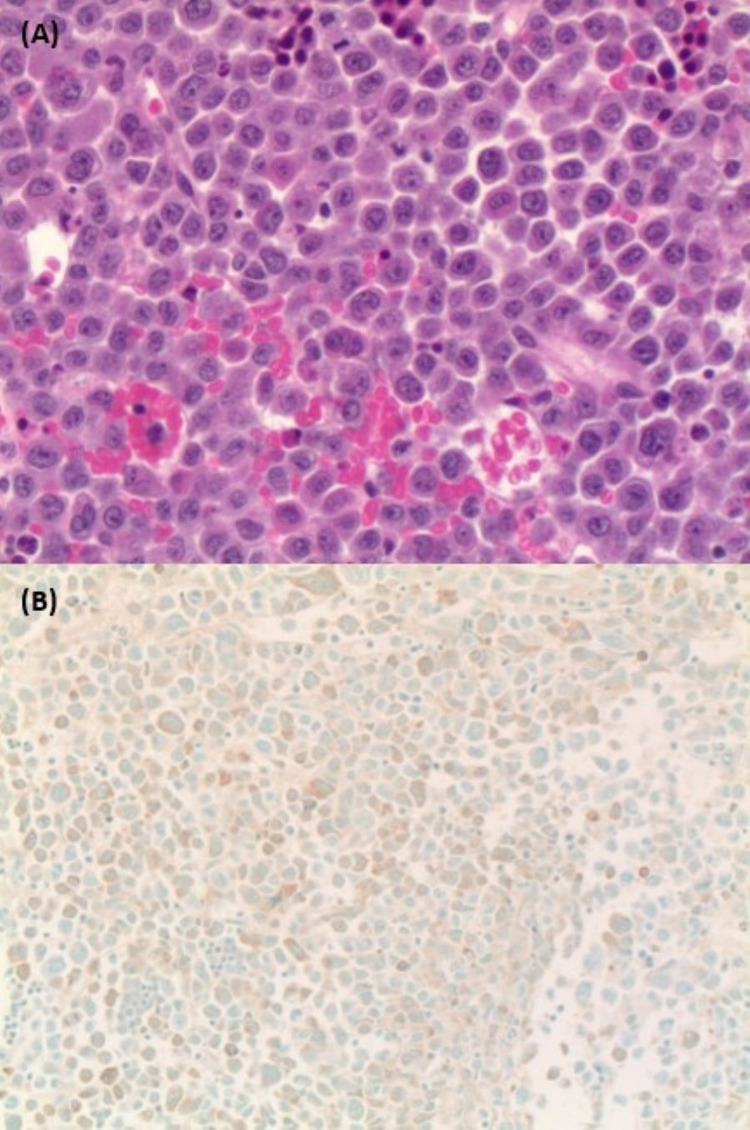
(A) Bone marrow biopsy hematoxylin and eosin stain showing sheets of blasts with large nuclei, prominent nucleoli, and a moderate amount of pink cytoplasm. (B) Immunohistochemistry showing blast cells staining positive for myeloperoxidase.

A 62-year-old man with a past medical history of Crohn’s disease, chronic alcoholism, alcoholic cirrhosis, deep vein thrombosis on apixaban, and hypertension presented to the emergency department with acute abdominal pain and decreased oral intake. Initial laboratory work showed a new leukocytosis with a WBC count of 28 K/µL, increased C-reactive protein (CRP) of 16.6 mg/dL, and increased erythrocyte sedimentation rate (ESR) of 37 mm/hr. The remainder of the initial lab work including hepatic function studies, coagulation studies, and lactic acid levels were within normal limits. CT of the abdomen and pelvis showed severe colitis involving the descending and sigmoid portions without evidence of obstruction. The patient was treated for acute Crohn’s colitis flare with antibiotics, corticosteroids, intravenous fluids, and analgesics. The gastroenterology team was consulted, and sigmoidoscopy was performed three days after admission, which showed severe inflammation of the descending and sigmoid colon with areas of edema, erosions, erythema, friability, mucous, and deep ulcerations. Several biopsies were taken showing focal ulceration and granulation tissue. The following day, the patient endorsed worsening abdominal pain. Repeat CT of the abdomen/pelvis showed new pneumoperitoneum, concerning possible iatrogenic colonic perforation. General surgery was consulted, and the patient was taken urgently to the operating room. He underwent exploratory laparotomy, subtotal colectomy, and end-ileostomy formation. The patient tolerated the procedure with no immediate complications. He was taken to the SICU post-operatively for further management. On POD 5, the patient endorsed continued abdominal pain with a worsening WBC count to 33 K/µL and decreased ostomy output. A CT angiography of the abdomen/pelvis was performed, which showed increasing pneumoperitoneum, large-volume ascites, and new thromboses in the superior mesenteric vein (SMV), splenic vein, and main portal vein. The patient was taken back to the operating room on POD 6 for re-exploratory laparotomy, drainage of abdominal ascites, abdominal washout, and wound vacuum placement. The visualized bowel was congested but viable; this was thought to be related to the new SMV thrombosis, so the patient was started on a continuous heparin infusion. The patient spent the next several weeks in the hospital recovering, and his hospital course remained tenuous. Although the patient remained alert and oriented, he displayed intermittent episodes of confusion. This was thought to be pain-related, but his encephalopathy persisted despite the use of a patient-controlled analgesia pump. On POD 22 from the re-exploratory laparotomy, the patient developed worsening encephalopathy and required intubation due to acute hypoxic respiratory failure likely related to aspiration of oral secretions, and ventilator settings were set in accordance with a lung-protective ventilation strategy. The patient’s clinical status acutely declined further into a shocking state, and he required multiple vasopressors to maintain his mean arterial pressure above 65 mmHg. Furthermore, repeat lab work showed an acute increase in WBC count to 69 K/µL. Hematology was consulted for a new hyperleukocytosis, and an urgent bone marrow biopsy of the right iliac crest was obtained by interventional radiology. The dark, maroon-colored stool was noted in the ostomy bag the following day, and the heparin infusion was discontinued. Despite appropriate conservative measures, the patient’s clinical status deteriorated further. He developed progressive multi-organ system failure. The family decided to pursue comfort care measures, and he passed away 24 days after the initial presentation. Three days later, the final bone marrow aspirate pathology revealed AML (Figures 3A, 3B).

## Discussion

CNS involvement is rarely seen in adult patients with AML while it is indeed more common in pediatric patients with AML [4]. Given this, very little is known about the impact of CNS involvement on prognosis and disease progression. Furthermore, the exact pathogenesis is incompletely understood. Several mechanisms for CNS involvement have been proposed, including calvarium bone marrow involvement and extension into bridging veins, involvement of the CSF, direct invasion into the brain parenchyma through the blood-brain barrier, and CNS hemorrhage with blood containing blasts [4]. In 2017, Alakel et al. performed an analysis of the largest cohort of AML patients to date. In this study, only 21 of the 3261 adult patients with confirmed AML were found to have CNS involvement, resulting in a prevalence of 0.6% [5]. Of these 21 patients, 18 had de novo AML. Compared to patients without CNS involvement, those who had CNS involvement had statistically significant higher levels of lactate dehydrogenase, higher prevalence of extramedullary AML, higher prevalence of FTL3-internal tandem duplication (ITD) mutation, higher prevalence of complex aberrant karyotypes, and higher rates of AML relapse [5]. Overall, the presence of CNS involvement in AML patients results in a worse prognosis. Since diagnostic lumbar puncture is rarely performed in asymptomatic patients, the overall incidence of CNS involvement in adult patients with AML may be underestimated. A diagnostic lumbar puncture can show blast cell counts ranging from as low as 5 cells/uL to higher than 1000 cells/uL. Additional non-specific findings include moderately elevated protein and glucose levels in the CSF. Flow cytometry of the CSF can also confirm the diagnosis. However, patients with neurological clinical manifestations can rarely show benign CSF studies without blast cells in the CSF, which was the cellular profile consistent with our first patient. The diagnostic imaging of choice is MRI, which can show various findings including intracerebral myeloid sarcoma, meningeal AML, or simply inflammatory enhancement [5,6]. This differs greatly from our first patient’s MRI findings, which showed abnormal bone marrow intensity signal and no parenchymal abnormalities. Early detection of CNS involvement in patients with newly diagnosed AML is paramount, as treatment usually involves the administration of intrathecal chemotherapy [5,6].

Furthermore, there is very little data in the literature regarding surgical stress as a risk factor for the development of AML. In fact, there are only two peer-reviewed published case reports describing the onset of AML following routine surgery. Ubukata et al. reported a case of AML that developed after surgery for advanced gastric cancer [7]. The patient in question was a 72-year-old man who underwent total radial gastrectomy for stage IIIB moderately differentiated gastric adenocarcinoma. There were no immediate periprocedural complications, and his postoperative course was unremarkable. Routine postoperative lab work revealed immature WBCs on a peripheral smear that progressively worsened over the following four weeks. The patient underwent a bone marrow aspiration biopsy five weeks after the surgery, which revealed 27.6% blast cells, and he was diagnosed with AML. Of note, the patient did not receive any adjuvant chemotherapy or radiation therapy perioperatively. This unusual case highlights the possibility of surgical stress potentially contributing to the development of de novo AML or possibly triggering acute blast crisis in a patient with previously undiagnosed chronic myeloid leukemia (CML).

Drury et al. reported a case of AML in a previously healthy 70-year-old man that developed after coronary artery bypass grafting [8]. This patient developed rapid onset multiorgan system dysfunction one day after surgery, including acute renal failure and acute respiratory distress syndrome requiring intubation and mechanical ventilation. It was theorized that a systemic inflammatory response resulting from the surgical trauma may have triggered the dramatic leukocytosis, with the patient’s WBC reaching as high as 91.7 L/µL. In retrospect, it is possible our two patients had undiagnosed CML, and the surgical stress resulting in a cascade of systemic inflammation may have triggered a transformation to AML. However, neither patient had prior hematological workups to suggest this underlying diagnosis. A better understanding of the inflammatory response to surgery may help to better risk stratify these patients prior to surgery. Regardless, the exact mechanism of surgical stress and the ensuing inflammatory response potentially leading to AML blast crisis is poorly understood, and more data is needed.

## Conclusions

AML is a rare and aggressive malignancy that can present with a broad range of clinical manifestations. CNS involvement is rarely documented, and clinicians should have a low threshold for performing lumbar puncture and/or MRI if clinically indicated, as confirmed CNS involvement may alter the treatment course and overall prognosis. Furthermore, little is known about the impact of surgical stress on the development of AML. Surgeons should be aware of this potential outcome following surgery, particularly if a leukemoid reaction develops post-operatively, as early detection can prevent delays in appropriate treatment. Further data are needed to better understand the pathogenesis and underlying inflammatory cascades following surgical trauma that possibly contribute to the development of AML.
